# Spatial Diversity of Bacterioplankton Communities in Surface Water of Northern South China Sea

**DOI:** 10.1371/journal.pone.0113014

**Published:** 2014-11-17

**Authors:** Jialin Li, Nan Li, Fuchao Li, Tao Zou, Shuxian Yu, Yinchu Wang, Song Qin, Guangyi Wang

**Affiliations:** 1 Key Laboratory of Coastal Biology and Bioresource Utilization, Yantai Institute of Coastal Zone Research, Chinese Academy of Sciences, Yantai, China; 2 Key Laboratory of Experimental Marine Biology, Institute of Oceanology, Chinese Academy of Sciences, Qingdao, China; 3 Key Laboratory of Coastal Environmental Processes and Ecological Remediation, Yantai Institute of Coastal Zone Research, Chinese Academy of Sciences, Yantai, China; 4 Graduate University of Chinese Academy of Sciences, Beijing, China; 5 Tianjin University Center for Marine Environmental Ecology, School of Environmental Sciences and Engineering, Tianjin University, Tianjin, China; 6 Department of Microbiology, University of Hawaii at Manoa, Honolulu, Hawaii, United States of America; National Taiwan University, Taiwan

## Abstract

The South China Sea is one of the largest marginal seas, with relatively frequent passage of eddies and featuring distinct spatial variation in the western tropical Pacific Ocean. Here, we report a phylogenetic study of bacterial community structures in surface seawater of the northern South China Sea (nSCS). Samples collected from 31 sites across large environmental gradients were used to construct clone libraries and yielded 2,443 sequences grouped into 170 OTUs. Phylogenetic analysis revealed 23 bacterial classes with major components *α-*, *β-* and *γ*-*Proteobacteria*, as well as *Cyanobacteria*. At class and genus taxon levels, community structure of coastal waters was distinctively different from that of deep-sea waters and displayed a higher diversity index. Redundancy analyses revealed that bacterial community structures displayed a significant correlation with the water depth of individual sampling sites. Members of *α-Proteobacteria* were the principal component contributing to the differences of the clone libraries. Furthermore, the bacterial communities exhibited heterogeneity within zones of upwelling and anticyclonic eddies. Our results suggested that surface bacterial communities in nSCS had two-level patterns of spatial distribution structured by ecological types (coastal VS. oceanic zones) and mesoscale physical processes, and also provided evidence for bacterial phylogenetic phyla shaped by ecological preferences.

## Introduction

The oceans harbor more than 3×10^28^ bacteria, which are organized within an estimated 10^6^ to 10^9^ taxa [1], [2]. These bacteria play vital roles in cycling nutrients and mediating climate on a global scale. However, bacterial communities in the oceans are structured by a variety of environmental factors, including currents, input of nutrients and pollutants, rising atmospheric carbon dioxide, and climate change [3], [4]. Although many studies have focused on the spatial and temporal diversity of marine bacteria, their responses to environmental perturbation remain undiscovered in many oceanic regions. The International Census of Marine Microbes (ICOMM) and the Global Ocean Sampling program have made tremendous efforts to obtain both basic and global information on marine bacterial diversity. However, information gaps remain to be filled in many ocean regions, particularlyin continental shelf regions such as coastal and marginal seas [5]. Clearly, in order to understand how environmental variables alter the community structure of microbial flora, it is necessary to proceed with broader research on bacterial communities and their distribution in relation to environmental conditions [6].

Marginal seas, major areas of biogeochemical cycling, are biologically more active regions compared with open oceans of the same latitude [7]. Meanwhile, these areas are characterized by their close relationships with adjacent terrestrial anthropogenic contaminations. The northern South China Sea (nSCS) is one part of the largest marginal sea located in the subtropical and tropical western North Pacific Ocean. It includes deep basins, with depths of over 5000 m, and the continental shelf; less than 100 mdeep. In summer, multi-scale physical processes mainly driven by the monsoon winds feature the nSCS with complex circulation as upwelling, costal currents, and cyclonic eddies [8]. The main body of nSCS water is oligotrophic, which is characterized with low nutrient concentrations, low phytoplankton biomass and low primary production [9]. Nutrient-rich fluvial input from the Pearl River discharges into the estuarine and adjacent waters, forming the sharp physical and chemical gradients over a small spatial scale [10]. Complex geographic and chemical marine systems make the nSCS sharing abundant biological diversity [11]. Thus, it provides an ideal area to investigate bacterial phylogenetic lineages shaped by environmental gradients from coast to open ocean, and from eutrophic and oligotrophic ecosystems [12], [13]. To date, most microbiological studies in the nSCS have focused on microbial resources and their applications [11], [14], and some studies on distribution of certain functional microbiota. Of microbial ecological studies in the nSCS [13], [15], [16], only a few reports investigated the relationship between bacterial abundance with water masses and nutrient status [17], [18]. Particularly, reports on the molecular characterization of bacterial communities in the surface water of the nSCS are rare and no studies have been carried out to investigate the effect of regulation of hydrologic variables on bacterial populations in this region.

This work aimed to describe spatial distribution patterns of bacterial communities in the nSCS surface water through analysis of samples collected from 31 sites. The sampling sites covered major environmental features, including the Pearl River estuarine, coastal, offshore, deep-sea, upwelling, and prospective eddies areas. We aimed to answer the following questions related to bacterial communities in the nSCS. What is the spatial diversity in the region? What are the major environmental factors shaping the community structure? It represents the first report of surface water bacterial communities in the nSCS.

## Materials and Methods

### Description of Study Area

**Figure 1:**
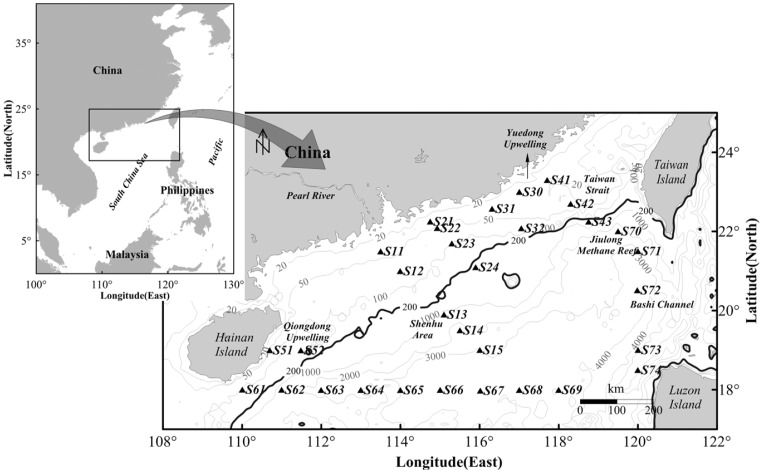
Location of the sampling sites in the nSCS (Isobath contour ploted by data from GEBCO website: www.gebco.net).

The nSCS is located south of the Tropic of Cancer and is heavily influenced by the East Asian monsoon system. It is connected with the East China Sea in the northeast through the Taiwan Strait, with the Pacific Ocean and the Sulu Sea in the east through Bashi Channel. The topography is characterized by a wide continental shelf and deep basins with maximum depth of 5,000 m at the center, and isobaths is parallel to the continental coastline. With large amounts of nutrient input from the Pearl River, and with fresh waters predominantly flowing along the coast via the coastal currents system, the nSCS features a gradient of P limitation in the estuary to N limitation in oceanic ocean [20]. Upwelling and eddies are common mesoscale phenomena mainly due to the southwest monsoon in summer. There are two strong upwelling regions in the inshore areas, Yuedong Upwelling from Shantou coast to the Nanri Islands (S30 site) and Qiongdong Upwelling in the east of Hainan Island (S52 site). The anomalous anticyclonic circulation is found along the 18°N latitude (S61 to S69 sites). Sampling sites represent most of the typical environments of the nSCS (Figure 1) and were classified into coastal and oceanic groups based on the 200 m water-depth contour.

### Sample Collection and Environment Characteristics

Seawater samples were collected from a water depth of 4.0–4.8 m using a rosette of Niskin bottles attached to a CTD probe frame during an Open Cruise of R/V *Shiyan 3* in August of 2007 (Figure 1). No specific permissions were required for these locations and activities. No endangered or protected species were involved in the field work of this study. The specific location (i.e., GPS coordinates) of sampling sites is listed in Table S1. For bacterial analyses, 20 L of surface seawater were aseptically filtered through Millipore 0.22-µm Millipore filter. The resulting filtrate was sealed in airtight sterile plastic tubes and stored at −80°C until use.

Temperature, salinity and depth were recorded by a Neil Brown MKIII CTD. Nutrient analyses were done in the South China Sea Institute of Oceanology, Chinese Academy of Sciences (Figure S1).

Sea level height anomaly data over the same sample-period was derived from the AVISO (Archiving, Validation, and Interpretation of Satellite Oceanographic data) website. A merged and gridded satellite product was generated based on TOPEX/Poseidon, Jason 1, ERS-1 and ERS-2 data [21]. The velocity field derived from SLA assuming geostrophic balance:
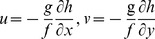



Where *h* is the SLA, *g* is gravitational acceleration, and *f* is the Coriolis parameter. Computational data were processed using MATLAB.

### DNA Extraction and Clone Library Construction

Total genomic DNA was extracted from the membrane filters of individual sites using standard phenol-chloroform extraction procedure described previously for filtrate material [22]. DNA was treated with RNase and subjected to two rounds of ethanol precipitation.

Fragments of 16S rRNA gene were amplified in a Tprofessional standard thermal cycler (Biometra) using bacterial universal primers 27F/1390R, under previously described PCR conditions [23]. PCR products were gel-purified, cloned into pGEM-T easy vectors (Promega), and then transformed into *Escherichia coli* TOP10 competent cells. Approximately, 120 colonies were randomly selected for sequence analysis. Plasmids carrying insert of correct size were sequenced using the SP-6 and T7 primers on an ABI model 3730 sequencer at Chinese National Human Genome Center (Shanghai, China).

The resulting sequences were aligned using Muscle v3.8 [24], then imported into Mothur v1.29 to remove chimera prior to further diversity analysis [25]. Sequences were classified using the mother Bayesian classifier (80% confidence) with the mothur-formatted version of the Ribosomal Database Project (RDP) training set (v. 9). The stand-alone BLAST v2.2.28 was used for local alignment of sequence similarity search with ‘env_nt’ databases in NCBI GenBank. The 16S rRNA gene sequences from each library with a percentage sequence identity of ≥97% were placed in the same Operational Taxonomic Unit (OTU). One representative sequence for each OTU was chosen to build a more concise phylogenetic tree using Mothur v1.29. The maximum likelihood tree was implemented in program PhyML v3.0 [26], on the basis of the best-fit substitution model as determined by *j*ModelTest v2.1 [27]. The 16S rRNA gene sequences were deposited in GenBank database under the accession numbers of KC872051–872789, KC872791–873358, KC873360–873759, and KC873761–874493.

### Diversity Comparison and Statistical Analyses

Diversity within each bacterial community (*α*-diversity) was assessed by plotting a rarefaction curve and calculating diversity indices, including Chao (*S_Chao_*) and the inverse Simpson index (*1/D*) using Mothur v1.29. In order to illustrate the scope of bacterial diversity, Good’s coverage (*C*) was calculated as [1-(*n/N*)] where *n* is the number of OTUs that had been observed once and *N* is the total number of OTUs in the sample.

Community comparison of bacterial assemblages (*β*-diversity) was performed with Fast UniFrac environmental clustering and principal coordinate analyses (PCoA) [28]. Diversity comparison matrix was generated into a heatmap based on the weighted UniFrac distance. Correlations between bacterial populations and environmental variables were determined by redundancy analysis (RDA) at class level by downweighting rare taxa in software Canoco v4.5 [29]. RDA was performed with the linear method because DCA (detrended correspondence analysis) on species variables revealed that the length of the first axis gradient was short (<2). Detrending was carried out in segments using the non-linear rescaling method. Prior to DCA and RDA, species values underwent square root transformation and environmental variables were normalized by z-score. The significance of the canonical axes was assessed using the permutation test with 499 unrestricted Monte Carlo permutations (P<0.05).

## Results

### Bacterial Diversity

To assess bacterial diversity, 2443 clones were selected from 31 bacterial libraries derived from surface water samples in the nSCS. Similarity of those sequences ranged from 65.7 to 100%. Non-redundant analyses identified 1,980 unique sequences, which were assigned into 310 OTUs. Of these sequences, 33% had less than 97% similarity with known sequences, which indicated that they were potential novel species. Moreover, three sequences (S51_38: KC823798, S51_47: KC873301, and S51_71: KC873325) had less than 95% identity with their best-matched reference sequences. About 67.3% of these sequences had their closest matches originally recovered from surface seawater collected along voyage from Eastern North American coast to the Eastern Pacific Ocean during the *Sorcerer II* Global Ocean Sampling Expedition [30].

The coverage of clone libraries ranged from 67.7 to 92.2% (Figure S2), suggesting that the selected sequences can reasonably represent bacterial communities of individual samples (Table 1). Of 31 sampling sites (Figure 1), sites S72 and S73 were observed with significantly higher bacterial diversity, S21 displaying the highest diversity found at site S21. Relatively low bacterial diversity was found at sites S32, S43 and S66 (*1/D* <2). Meanwhile, spatial variation of bacterial diversity was observed in the study area.

**Table 1:** Biodiversity indices of bacterial communities obtained from the sampling sites of the nSCS.

Sites	Sequences	*C* (%)	OTUs	*S_Chao_*	*1/D*
**S11**	67	88.06	17	22.6	5.91
**S12**	92	86.96	20	36.5	3.22
**S13**	92	88.04	19	37.3	3.76
**S14**	86	79.07	30	60.6	12.14
**S15**	71	76.06	26	53.2	7.22
**S21**	73	84.93	28	34.1	18.25
**S22**	89	87.64	28	33.0	11.32
**S23**	80	88.75	21	28.2	10.29
**S24**	88	92.05	13	23.5	4.64
**S30**	68	67.65	30	76.2	7.40
**S31**	73	80.82	24	46.8	9.25
**S32**	77	92.21	10	25.0	1.69
**S41**	83	86.75	23	32.2	8.49
**S42**	84	85.71	20	31.0	3.18
**S43**	83	91.57	10	31.0	1.41
**S51**	86	69.77	36	90.2	11.57
**S52**	66	78.79	22	67.5	9.84
**S61**	87	71.26	34	94.0	9.74
**S62**	74	74.32	29	57.5	9.19
**S63**	78	74.36	29	67.0	7.07
**S64**	80	88.75	14	23.0	1.83
**S65**	72	69.44	31	88.8	11.94
**S66**	82	91.46	11	21.5	1.54
**S67**	85	78.82	26	56.6	4.88
**S68**	70	74.29	25	76.0	9.66
**S69**	75	81.33	24	37.0	7.71
**S70**	62	79.03	19	58.0	7.95
**S71**	76	86.84	15	37.5	2.43
**S72**	79	69.62	30	168.0	9.22
**S73**	77	74.03	25	120.0	7.26
**S74**	88	90.91	15	24.3	2.27

### Phylogeny of Bacterial Community

**Figure 2:**
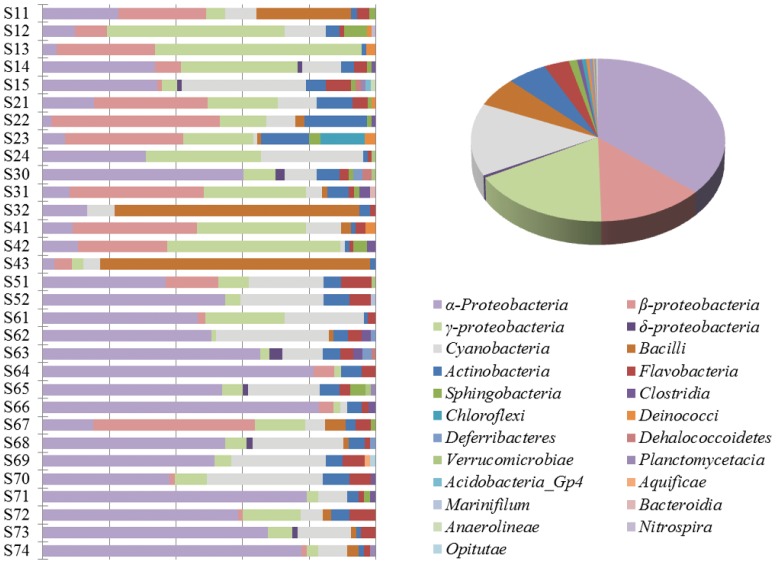
Phylotype distribution and comparison of the clone libraries from individual sampling sites (histogram) and entire nSCS (pie).

The vast majority (93.1%) of acquired sequences were affiliated with 4 bacterial phyla, *i.e.*, *Proteobacteria* (*α*-36.6%; *β*-16.6%; and *γ*-13.3%), *Cyanobacteria* (13.9%), *Bacilli* (6.9%), and *Actinobacteria* (5.8%). Overall, the identified sequences belonged to 23 bacterial classes, suggesting a great variety of bacterial communities in the study area (Figure 2). The phyla of *Proteobacteria*, *Acidobacteria*, *Deferribacteres*, *Planctomycetes*, and *Verrucomicobia* were shared with sediment samples from nSCS [31], [32]. *Alphaproteobacteria* and *Actinobacteria*, were observed in all sampling sites, and interestingly, members of *α-Proteobacteria* constituted more than 70% of 4 clone libraries derived from 4 sites (S64, S66, S71 and S74). *Bacilli* were the most dominant class at sites S43 (81.0%) and S32 (73.3%), *γ*-*Proteobacteria* at sites S12 (53.4%) and S13 (62.0%), and *β-Proteobacteria* at site S22 (50.6%). Rare bacterial classes, whose sequences were observed only once from a library, were identified as members of *Acidobacteria_Gp4* (S15), *Aquificae* (S69), *Bacteroidia* (S31), *Nitrospira* (S12), *Anaerolineae* (S15), and *Opitutae* (S69).

**Figure 3:**
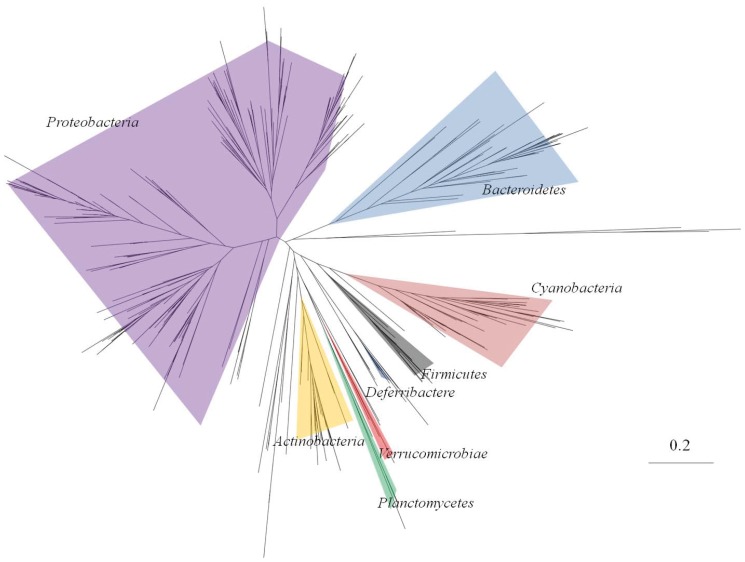
Maximum likelihood phylogenetic radual tree generated by 170 OTUs assembled of 2443 sequences derived from 31 bacterial clone libraries. The detailed polar tree was shown in Figure S3.

Classification analysis (80% confidence threshold) revealed that 170 OTUs belonged to members of *Proteobacteria* (Figure 3 and Figure S3). Those OTUs were distinctively clustered with *α-*, *β-*, *γ*- and *δ- Proteobacteria*. Of these OTUs, 83 fell into the class *α-Proteobacteria* and clustered with *Caulobacterales*, *Rhizobiales*, *Rickettsiales*, *Rhodospirillales*, *Rhodobacterales*, and *Sphingomonadales*. The OTUs, which were clustered with unclassified *α-Proteobacteria,* had close affiliations with sequences derived from Chesapeake Bay, coastal Delaware Bay and open sea Panama regions in the Pacific [33], [34]. Furthermore, 21 OTUs, which were members of *Burkholderiales* in the phylume *β-Proteobacteria*, were mostly affiliated with accelerating utilization of organic nitrogen. Members of *γ-Proteobacteria* contributed to major components of 12 phylogenetic clades in the nSCS bacterial libraries. Their abundance and rich diversity supports their important ecological functions, including anaerobic sulfur and ammonia oxidation [35]. An abundance of *Enterobacteriaceae*, which were best-matched with sequences isolated from the human gut, suggests that anthropogenic influence brought non-marine origins into nSCS microbiota. Notably, the genus *Alteromonas* sp., whose presence substantially promots growth of toxic dinoflagellate *Alexandrium fundyense* [36], was present in extraordinarily high concentrations at sites S14 and S61. Those two sites were located at the regions where algal blooms frequently occurred in summer [37], [38]. The remaining minority OTUs were members of the orders *Bdellovibrionales* and *Desulfobacterales* in the class of *δ-Proteobacteria,* which were reported to play a fundamental role in sulfur and metal element biogeochemical cycling [39].

Thirty-one OTUs were affiliated with the phylum *Cyanobacteria* (Figure 3). They were detected in almost all libraries except the library of S13 and best matched with sequences from a wide range of aquatic samples in Chesapeake Bay, Coco’s Island, Antarctica Lake Vida, Sargasso Sea and Panama Canal. GpIIa (*Synechococcu*) was the largest genus that clustered with culture-independent representative clones. GpXI (mostly members of *Microcystis* strains) and *Bacillariophyta* were minor components with located-specific distribution at Pearl River Estuary and continental area, respectively.

Forty OTUs belonged to *Bacteroidetes*. Those phylotypes fell into three classes (*Bacteroidia*, *Sphingobacteria* and *Flavobacteria*) and one unclassified group. The phylum *Bacteroidetes* has been implicated as a major utilizer of complex polymers and is abundant in marine ecosystem, especially in eutrophic waters [40]. It was concentrated at shallow-water site S51, close to Hainan Island (accounting for 10.9% of sequences).

Members of the other 8 phyla were also identified in nSCS. Some clades were site-specific, for example *Chloroflexus* of *Chloroflexi* (at S23) and *Enterococcus* of *Firmicutes* (at sites S11 and S32). Four sequences (S51_38: KC873293, S51_47: KC873301, S51_71: KC873325 and S61_67: KC873469) were distantly related to others and represented the deep-rooted branch (Figure S3), indicating that they are divergent from other bacteria in the library. Eleven sequences (S14_12: KC872313, S24_13: KC872712, S30_16: KC872802, S30_35: KC872817, S30_40: KC872821, S62_02: KC873497; S63_02: KC873571, S63_20: KC873586, S63_27: KC873593, S68_41: KC874004 and S72_48: KC874293) formed a monophyletic clade (Figure S3) and were closely related to other uncultured sequences that derived from seawater of Panama and Northeast subarctic Pacific Ocean [34], [41].

### Spatial Distribution and Diversity Comparison

**Figure 4:**
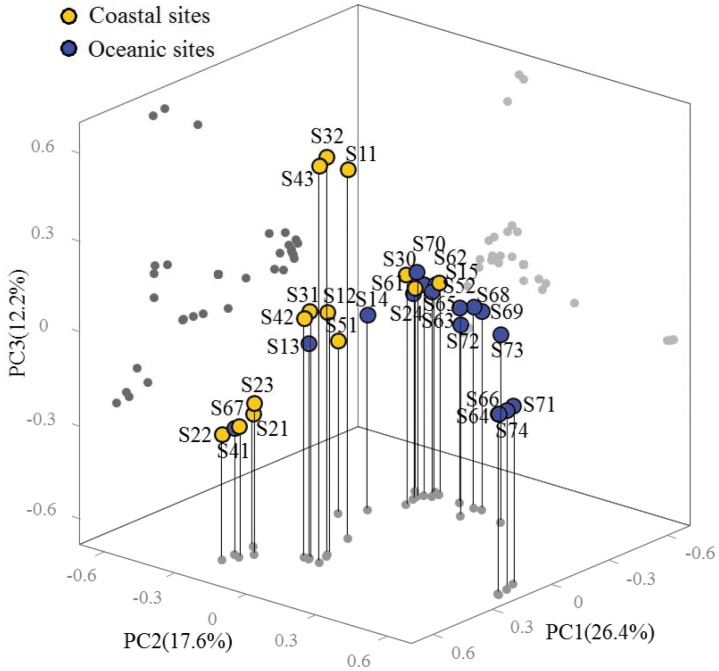
Ordination diagram for PCoA of the bacterial communities using weighted UniFrac with 16S rRNA sequences. Shown is the first three principal coordinate vectors (PC1, PC2 and PC3) and the distribution of bacterial libraries in response to these axes.

**Figure 5:**
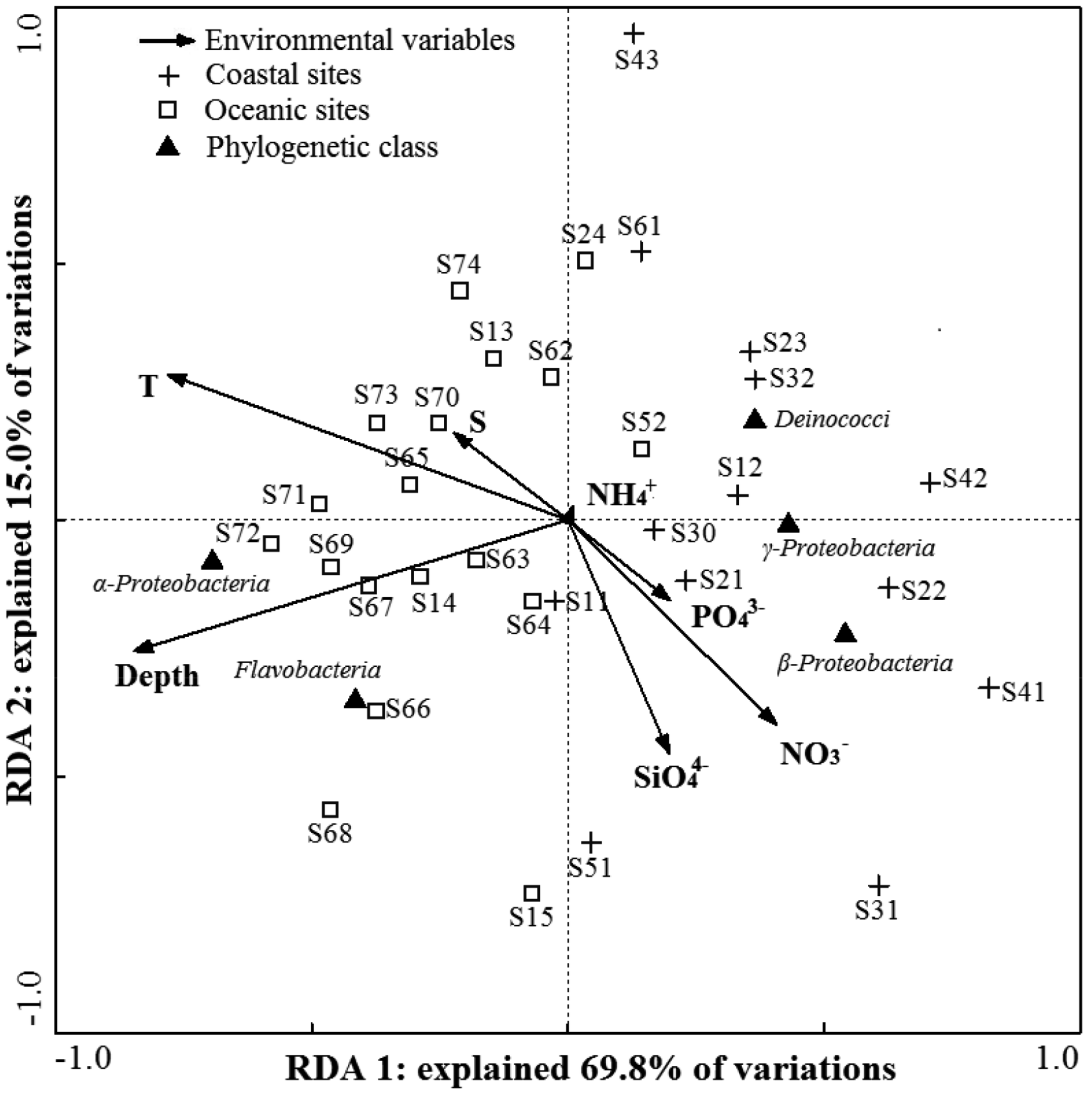
RDA ordination plots for the first two dimensions of the relationship among the sampling sites, environmental variables and bacterial phylogenetic classes. Environmental variables are represented by arrows with a cutoff of *r* ^2^ = 0.2. Correlations are indicated by the length and angle of arrows.

The maximum likelihood phylogeny was used to examine phylogenetic comparison between bacterial libraries using a UniFrac based method. The first three principal coordinates (PC) together accounted for 56.2% of the variation. Considering these primary vectors, the bacterial assemblages derived from sites close to continental shelf (water depth <200 m) were generally more similar amongst one another versus those from oceanic areas (water depth >200 m) with the exception of S30, S52 and S67 sites (Figure 4). Comparison between two individual bacterial communities revealed overall high distances, suggesting an underrated and versatile role of bacteria within various marine environments with a highly niche-specific community structure (Figure S4). Spatial distribution of surface-water bacterial assemblages might be influenced by a variety of hydrological and physio-chemical factors, such as ocean currents, thermohaline background, and eutrophication condition. RDA of bacterial classes was used to reveal their relationship with environmental variables (Figure 5). The sum of all canonical eigenvalues indicated 30.0% of the total variation can be explained by environmental variations. Concerning the bacterial class data, the first two RDA axes explained 25.4% of the total variance in the bacterial composition and accounted for 84.8% of the cumulative variance of the bacteria-environment relationship. Correlations of bacterial classes and environment variables were 66.0% and 53.2% for axis RDA 1 and 2, respectively.

RDA1 represented a depth gradient and had a correlation coefficient of −0.5589. It distinguished the bacterial assemblages derived from sites of continental shelf from those of oceanic sites. RDA2 represented a silicate gradient caused by silicate and had a correlation coefficient of −0.2419. Based on the partial Monte Carlo permutation test (P<0.05), the variable of the depth alone contributed significantly (*P* = 0.005, *F* = 5.28) to the bacteria-environment relationship, providing 50.0% of the total CCA explanatory power. Although no other variables had statistically significant contribution to the relationship, thermohaline background of temperature and salinity provided more RDA explanatory power on bacterial composition than nutrition concentration. The correlation of the bacterial classes with environmental variables indicated that *α-* and *β-Proteobacteria* were major components that contributed to statistical difference of cluster analyses between sites of coastal and oceanic sites. The distribution of *α-Proteobacteria* was positively correlated with the depth of sampling sites, while the distribution of *β-Proteobacteria* was positively correlated with the nitrate concentration and negatively correlated with salinity.

## Discussion

Studies on bacterial community composition in marine systems are nowadays routinely done by using culture-independent methods. For bacterial ecologists, it is tempting to correlate bacterial taxonomy and functions to particular environmental features. However, this relationship is far from conclusive because both samples and datasets are scanty relative to the vast bacterial categories and habitat types [42]. Particularly, the ocean is the largest contiguous environment and characterized by strong physical mixing of currents and storms, different nutrient factors, and occurrence of widely distributed microbes [2]. Characteristics of bacterial communities need to be approached and determined in more marine areas beyond the North Pacific [43], Arctic [44], and Mediterranean Sea [45]. Surface samples of nSCS water can be taken from diverse environmental habitats, such as coastal vs. oceanic, oligotrophication vs. eutrophication, and saline vs. freshwater [46]. This study is the first report of the surface seawater bacteria in a large environmental gradient, with variations in the oceanic province. Our results revealed that community structures appear to be spatial heterogeneity of distribution driven by habitat characteristics.

### Diversity and Novelty of Bacterial Assemblages

Diverse and novel bacterial species were observed in this study (Figure 2). Bacterial communities showed higher diversity than previous reports in the same region, and shared several phyla with sediment samples from nSCS [30], [31], [47]. A great part of the 16S bacterial dataset had their closest matched sequences originally detected from the *Sorcerer II* Global Ocean Sampling expedition that has reported the most extensive dataset of microbiota in surface water consisting of 7.7 million sequences [30]. These results indicated that similar habitats may contain a similar genetic diversity of bacterial communities. Compared with the sequences collected from the *Sorcerer II* expedition, the major phylum were *Proteobacteria*, *Cyanobacteria*, *Firmicutes*, *Actinobacteria*, *Bacteroidetes*, and *Planctomycetes*. The results of this study also indicated that nSCS contained the dominant surface-seawater bacterial groups (*α-Proteobacteria*, *γ-Proteobacteria* and *Cyanobacteria*) commonly found in other regions [48]. Nevertheless, a relatively high proportion of *β*-*Proteobacteria* was detected in the clone library, which is generally found in small proportions (approximately <3%) in other oceanic surface seawaters [43], [44], [49].

Some sequences were found to be novel at species level and even at order level. Most of these species were collected from the coastal area of nSCS, especially at the S51site, which is the center of Qiongdong Upwelling. The anthropogenic activities of coastal urbanization, industrialization and economic growth have led to the current pollution through the increasing input of metal contaminants, nutrient substances and organic carbon in the last few decades [38]. Coupled with the upwelling system at southeast of Hainan Island, S51 was characterized by low temperature, high salinity, low dissolved oxygen, high chlorophyll *a* and primary production [50]. Furthermore, different from another Yuedong Upwelling, seawater of Qiongdong Upwelling is also enriched with silicon [51]. The unique temperature, salinity and silicon concentration were also detected during sampling period (see Figure S1). These physiochemical variables may have contributed to the formation and evolution of new microbial species [52].

### Environmental Influence on Bacterial Distribution Pattern

Based on the bacterial communities clustering analysis at species level, the bacterial communities of oceanic sites had more commonalities than those in coastal sites (Figure 4), which was further reflected at phylum level (Figure 5). Bacterial diversity index (*1/D*) revealed a generally inverse relationship to depth of sites along all transects (Table 1), suggesting that bacterial community was more diverse in coastal area. Spatial distances did not generate considerable differences in bacterial community composition, which likely resulted from contiguous environments due to physical mixing of currents and storms [46]. Furthermore, bacterial distribution patterns showed large-scale continuum and beta-diversity heterogeneity through intermediate habitat types across coastal and oceanic ecosystems. This is consistent with what has been reported from the synthesis of global and pole bacterial datasets [49], [53].

Large proportion of sequences belonged to the members of *α-Proteobacteria*, supporting the dominance of *α-Proteobacteria* in saltwater [54]. Moreover, a significantly high percentage of *α-Proteobacteria* (*P* = 0.000, *F* = 15.572) in coastal water communities concurred with a previous report on global ocean sampling data [52]. *SAR11*, the most abundant free-living cluster, was also found in this study (Figure S5). The increase of SAR11 relative abundance in oceanic samples corresponded well with local oligotrophic conditions and also supported previous reports [55].

It was unexpected that the depth of sampling sites as the principal factor determines bacterial community structure in the surface water. Previous studies have revealed the existence of bacterial variation between coastal and oceanic seawaters [48]. As depth was unlikely to directly impact surface water, the most proper explanation was that the variation in bacterial populations was due to synergetic driving forces of environmental variables, which are involved in characteristics of coastal and oceanic waters. In other words, the different habitats (reflected in water depth) should account for variability in bacterial community composition. On the other hand, depth of sample sites seemed to have little impact on oceanic bacterial community distribution as it was an only factor, which was correlated with spatial distance [48]. Nutrients were originally expected to play a major role in the microbial composition based on the shift from P to N limitation in nSCS [10]. Nevertheless, 4 parameters of nutrients only explained 7% bacterial variability, which could be explained by the limited impacts of scale and scope on surface water transmitting by coastal currents from terrestrial input [56]. All the environmental variables can only explain 30% of the variability in the community composition. Thus, the composition of bacterial assemblages was additionally influenced by other environmental variables not investigated in the present study, such as residence time, availability of metal elements and bacterial competitors (e.g., protists, viruses and metazoans).

**Figure 6:**
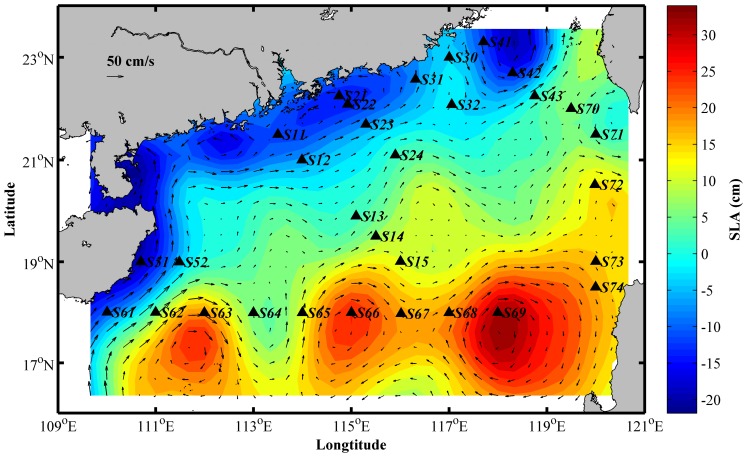
Sea level anomaly derived from the sea level anomaly maps during August 2007 provided by the AVISO.

Previous studies have revealed that the physical oceanographic processes influence phytoplankton stocks and production by monsoon-driven circulation and upwelling in the SCS [18], [57]. Moreover, bacterial assemblages were proved to be distinct in order to adapt for different oceanographic water masses in eastern Australian sea [38]. In our study, the bacterial community structures were apparently discrete in several sites of similar geochemical conditions. It is likely that the hydrological factors lead to this dissimilarity by stimulating the existence of clusters belonging to adjacent areas or layers through transportation (Figure 4). The abnormal community diversity was observed at the sites S30 and S52 with higher similarity to deep-sea samples, which might be influenced by the upwelling system of Qiongdong and Yuedong, respectively. The future study of bacterial diversity throughout water profile would demonstrate whether this difference was generated by insertion of components from lower layer. Moreover, community structure appeared discrete at the sites S61 to S69. The existence of mesoscale local anticyclonic circulations in summer has been reported [8]. The data from AVISO also proved their existence during sampling period (Figure 6). It is likely that mass transport or extraordinarily hydrology of special current systems could result in the opportunistic taxa and the ecological shift of bacteria in surface seawater.

In conclusion, the composition of bacterial communities exhibited remarkable biogeographic differences between coastal and oceanic ecological systems in surface seawater of nSCS. Similar to other coastal environments, bacterial communities were dominated by members of *Proteobacteria*, *Cyanobacteria*, and *Bacteroidetes.* Moreover, bacterial communities derived from upwelling and mesoscale anticyclonic eddy sites displayed abnormal compositions compared with those of adjacent sites (Figure 4 and Figure S4). Our finding of spatial heterogeneity in marine contiguous environment implied that environmental factors other than dispersal (?) were the drivers of the distribution of bacterial compositions. This study demonstrated that bacterial composition at class level was influenced by the depth of sampling sites. Further investigation to define biomes for underlying patterns of marine bacteria should focus on what common rules of natural selection impact the bacterial communities and how bacteria change the functional biogeochemical cycle.

## Supporting Information

Figure S1 **Contour maps of environmental variables in nSCS.**
(TIF)

Figure S2 **Rarefaction curve of 16S rRNA clone libraries derived from nSCS.** Phylogenetic diversity is represented by branch length.
(TIF)

Figure S3 **Maximum likelihood phylogenetic polar tree generated using 170 OTUs.**
(TIF)

Figure S4 **Heatmap showing the bacterioplankton diversity comparison among different sites.** The scale at the bottom of the heatmap indicates the similarity level between each comparison. The darker the color is, the more different the two comparing bacterioplankton communities are.
(TIF)

Figure S5 **Phylogenetic tree of the 16S rRNA clusters affiliated with the α-Proteobacteria lineage, constructed from an alignment of OTUs from nSCS in bold.** Reference sequences were selected from GenBank with accession numbers are in parentheses. The OTU names were labeled with the numbers of contained sequences, while were designated as sequence name when containing only one sequence.
(TIF)

Table S1 **Coordinates and characteristics of the sampling sites.**
(DOCX)
