# Supplementary figures and images for: Spatial Diversity of Bacterioplankton Communities in Surface Water of Northern South China Sea
Source: PLoS One. 2014 Nov 17;9(11):e113014. doi: 10.1371/journal.pone.0113014 (PMC4234503; doi:10.1371/journal.pone.0113014)

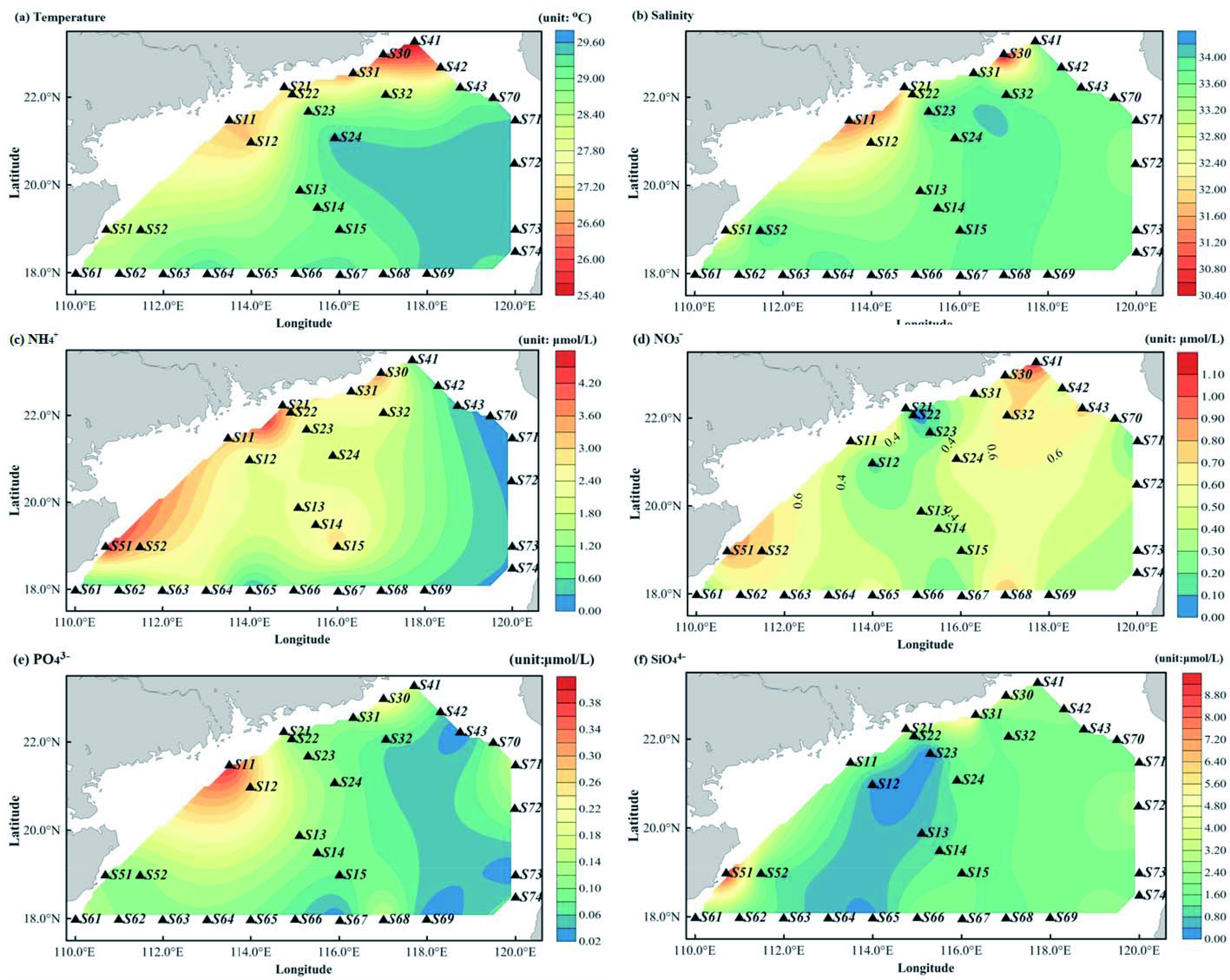

Supplement: Figure S1 — Contour maps of environmental variables in nSCS. (TIF) [file pone.0113014.s001.tif]

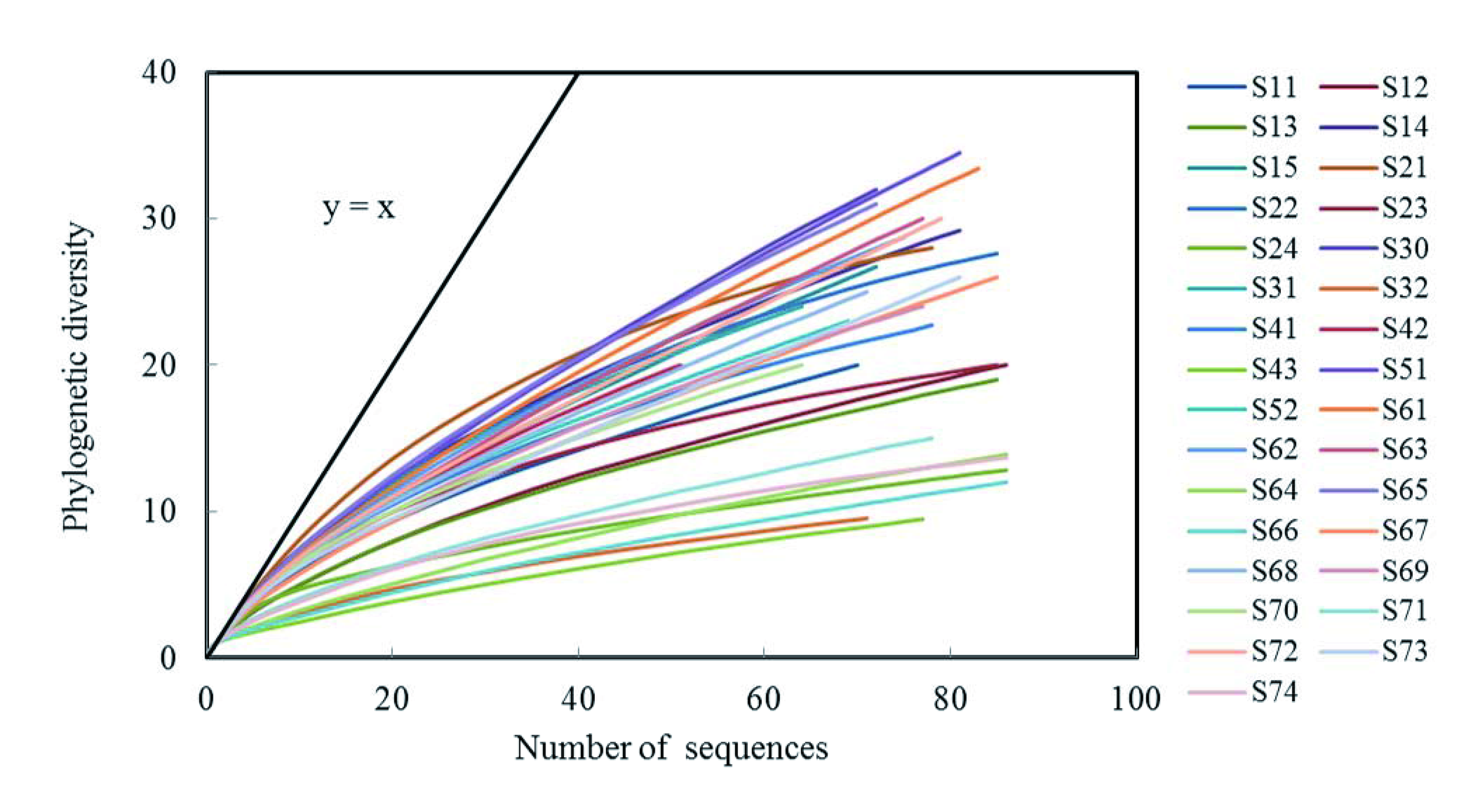

Supplement: Figure S2 — Rarefaction curve of 16S rRNA clone libraries derived from nSCS. Phylogenetic diversity is represented by branch length. (TIF) [file pone.0113014.s002.tif]

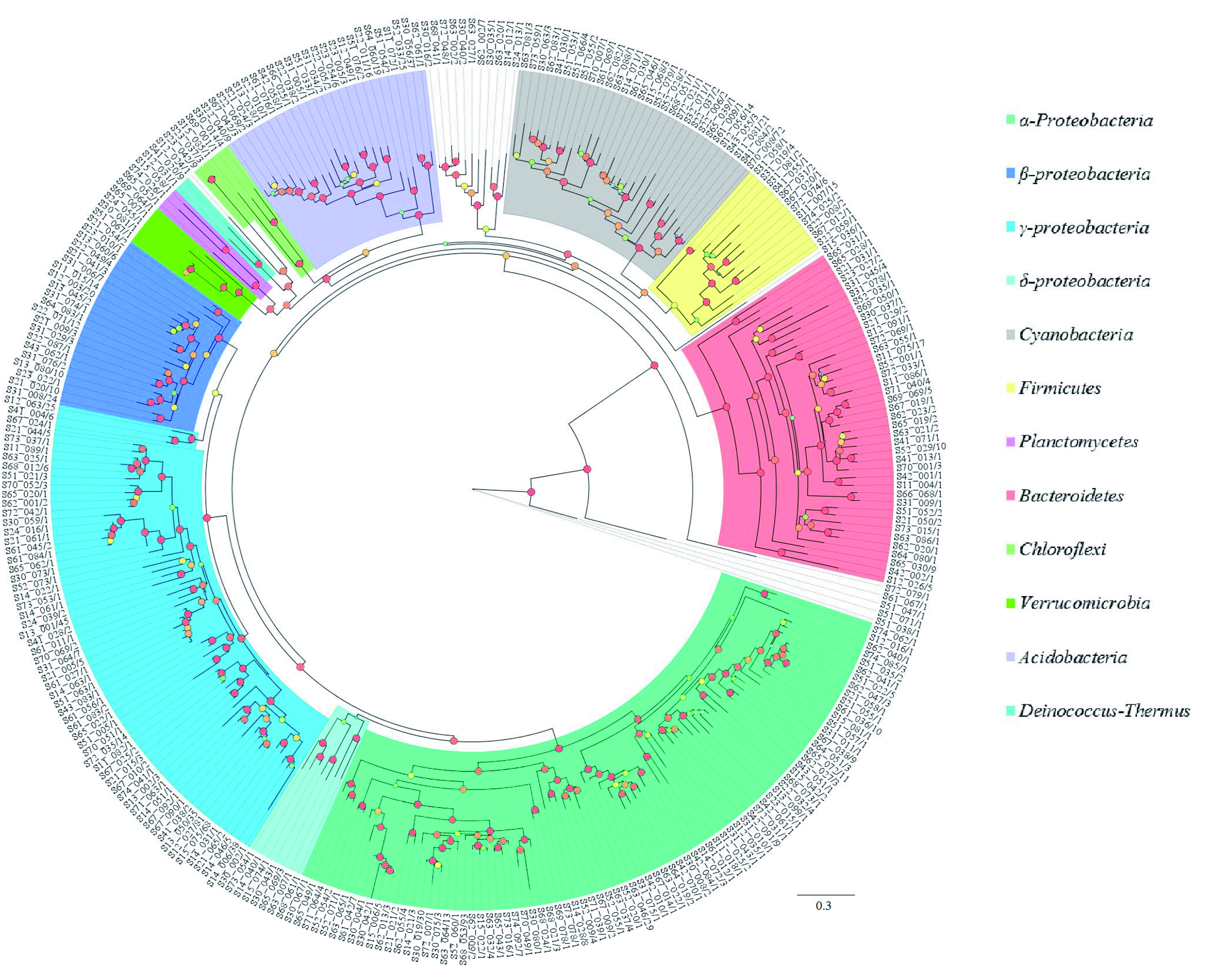

Supplement: Figure S3 — Maximum likelihood phylogenetic polar tree generated using 170 OTUs. (TIF) [file pone.0113014.s003.tif]

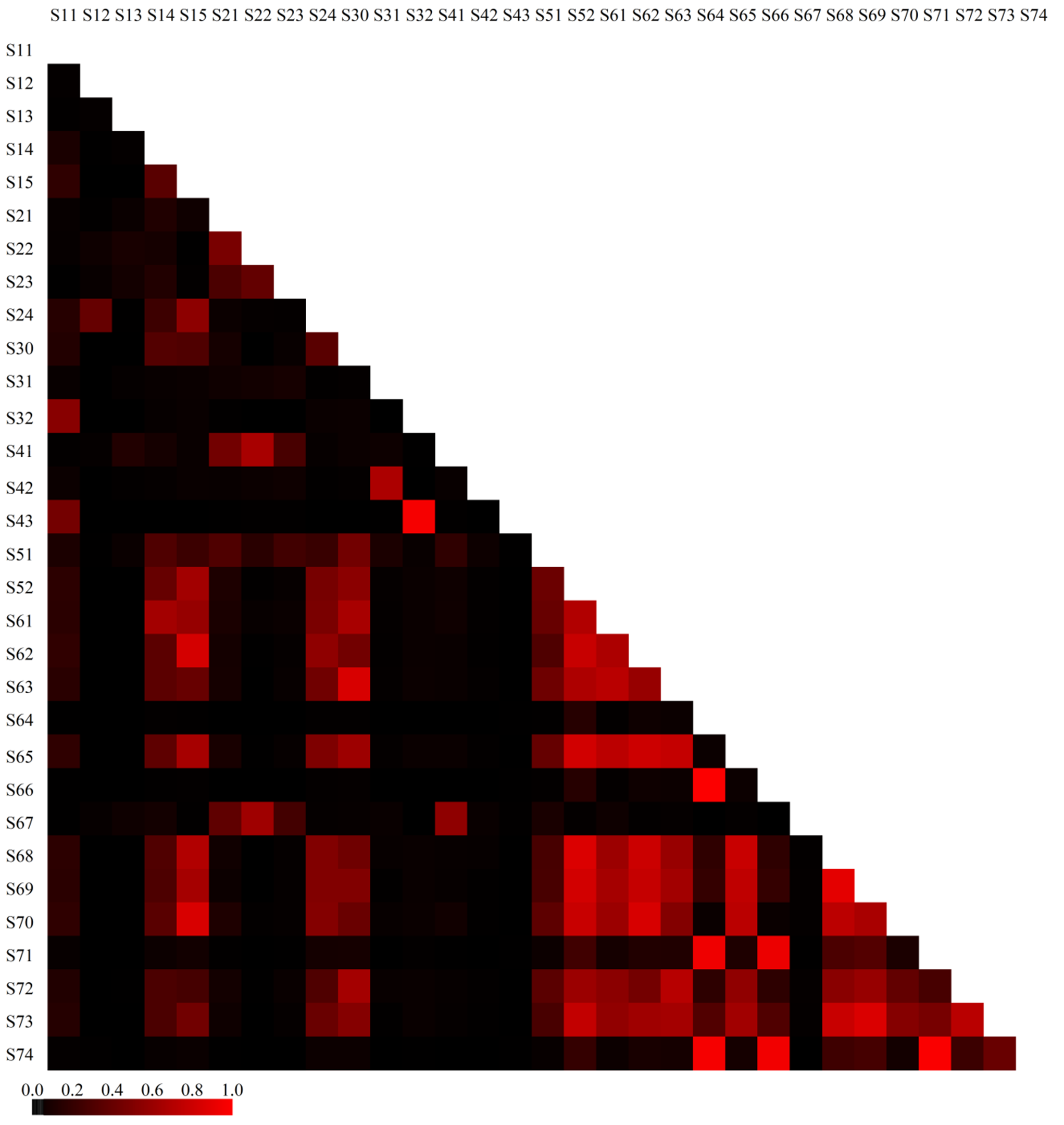

Supplement: Figure S4 — Heatmap showing the bacterioplankton diversity comparison among different sites. The scale at the bottom of the heatmap indicates the similarity level between each comparison. The darker the color is, the more different the two comparing bacterioplankton communities are. (TIF) [file pone.0113014.s004.tif]

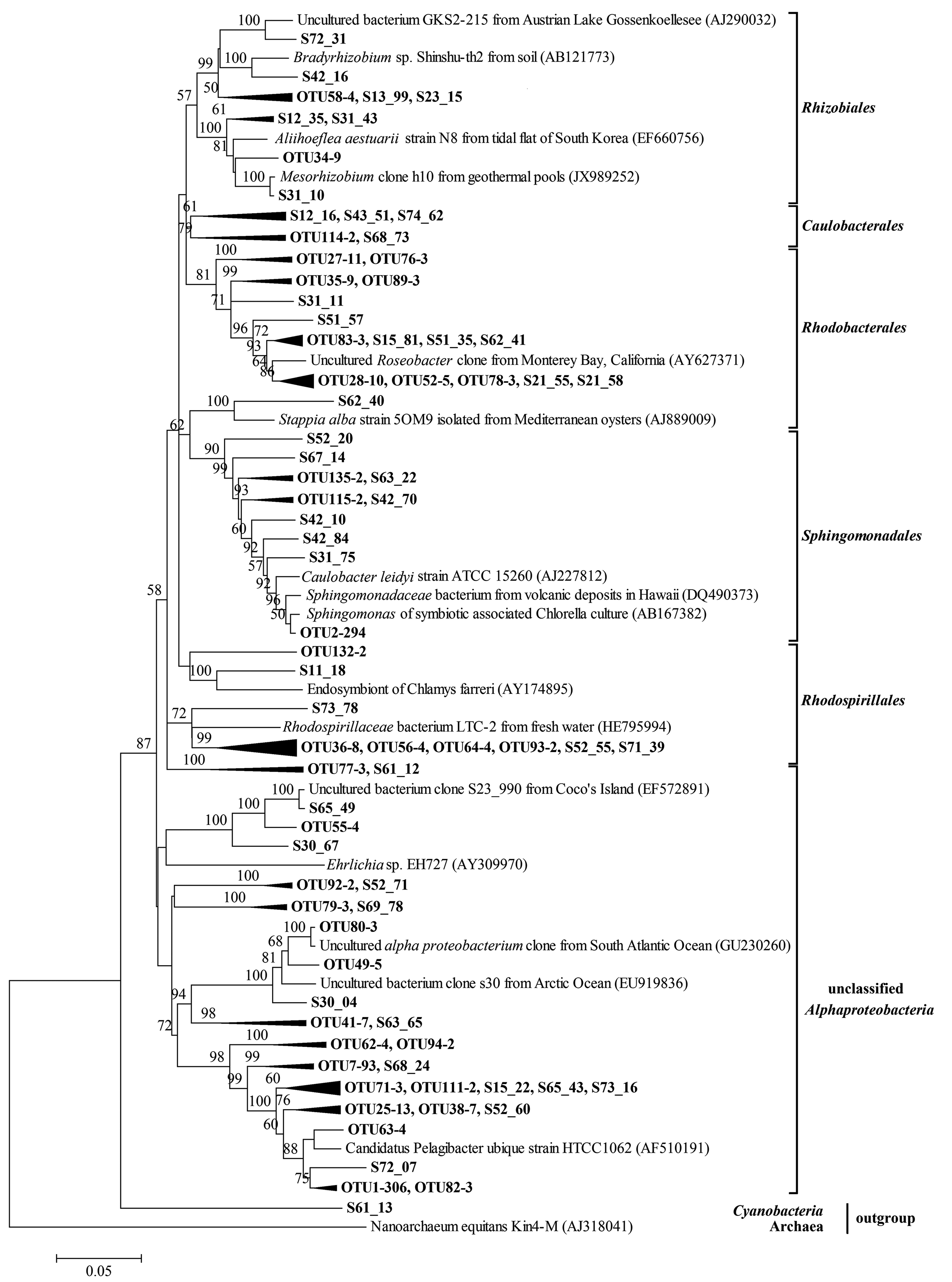

Supplement: Figure S5 — Phylogenetic tree of the 16S rRNA clusters affiliated with the α-Proteobacteria lineage, constructed from an alignment of OTUs from nSCS in bold. Reference sequences were selected from GenBank with accession numbers are in parentheses. The OTU names were labeled with the numbers of contained sequences, while were designated as sequence name when containing only one sequence. (TIF) [file pone.0113014.s005.tif]
