# Supplementary material for: Spatial Diversity of Bacterioplankton Communities in Surface Water of Northern South China Sea
Source: PLoS One. 2014 Nov 17;9(11):e113014. doi: 10.1371/journal.pone.0113014 (PMC4234503; doi:10.1371/journal.pone.0113014)
Supplement: Table S1 — Coordinates and characteristics of the sampling sites. (DOCX) [file pone.0113014.s006.docx]

**Table S1.** Coordinates and characteristics of the sampling sites.

| Sites | Latitude  (°N) | Longitude  (°E) | Depth  (m) | Environmental types |
| --- | --- | --- | --- | --- |
| S11 | 21.50 | 113.50 | 40 | Coast, river mixing zone |
| S12 | 21.00 | 113.99 | 84 | Coast, river mixing zone |
| S13 | 19.90 | 115.10 | 1122 | Ocean |
| S14 | 19.50 | 115.50 | 2411 | Ocean |
| S15 | 19.01 | 116.01 | 3154 | Ocean |
| S21 | 22.25 | 114.75 | 34 | Coast, river mixing zone |
| S22 | 22.09 | 114.93 | 55 | Coast, river mixing zone |
| S23 | 21.70 | 115.30 | 102 | Coast |
| S24 | 21.10 | 115.90 | 299 | Ocean |
| S30 | 23.00 | 117.00 | 36 | Coast, up-welling center, river mixing zone |
| S31 | 22.57 | 116.32 | 49 | Coast |
| S32 | 22.08 | 117.05 | 75 | Coast |
| S41 | 23.30 | 117.71 | 27 | Coast |
| S42 | 22.70 | 118.30 | 34 | Coast |
| S43 | 22.25 | 118.75 | 145 | Coast |
| S51 | 19.00 | 110.70 | 65 | Coast, river mixing zone |
| S52 | 19.00 | 111.48 | 154 | Coast, up-welling center |
| S61 | 18.00 | 110.00 | 97 | Coast, anticyclonic circulation |
| S62 | 18.00 | 111.00 | 1484 | Ocean, anticyclonic circulation |
| S63 | 18.00 | 112.00 | 2456 | Ocean, anticyclonic circulation |
| S64 | 18.00 | 113.00 | 2157 | Ocean, anticyclonic circulation |
| S65 | 17.99 | 114.00 | 3241 | Ocean, anticyclonic circulation |
| S66 | 18.00 | 115.00 | 3807 | Ocean, anticyclonic circulation |
| S67 | 17.98 | 116.02 | 3956 | Ocean, anticyclonic circulation |
| S68 | 18.00 | 117.00 | 4036 | Ocean, anticyclonic circulation |
| S69 | 18.00 | 118.00 | 3988 | Ocean, anticyclonic circulation |
| S70 | 22.01 | 119.50 | 2441 | Ocean |
| S71 | 21.50 | 120.00 | 3017 | Ocean |
| S72 | 20.52 | 119.98 | 3470 | Ocean |
| S73 | 19.01 | 120.00 | 3258 | Ocean |
| S74 | 18.50 | 120.00 | 1865 | Ocean |
